# Controllable Preparation of Highly Crystalline Sulfur-Doped Π-Conjugated Polyimide Hollow Nanoshell for Enhanced Photocatalytic Performance

**DOI:** 10.3390/polym15040903

**Published:** 2023-02-11

**Authors:** Duoping Zhang, Chenghai Ma, Peidong Shi, Zuan Yang, Tongwei Rong, Liurui Xiong, Wenhui Liao

**Affiliations:** 1 School of Chemical Engineering, Qinghai University, Xining 810016, China; 2 Department of New Energy (Photovoltaic) Industry Research Center, Qinghai University, Xining 810016, China

**Keywords:** porous hollow shell, sulfur-doped polyimide, solar light, degradation of MO

## Abstract

In this study, a series of highly crystalline π-conjugated polyimide photocatalysts with porous nano hollow shell (HSPI) was prepared for the first time by the hard template method by adjusting the addition ratio of the template precursor. SiO_2_ nanospheres not only serve as template agents but also as dispersants to make precursors of SPI more uniform, and the degree of polymerization will be better, resulting in significantly enhanced crystallinity of HSPI relative to bulk SPI (BSPI). More strikingly, it is found that HSPI has a larger specific surface area, stronger visible light absorption, and higher separation efficiency of photogenerated electron and hole pairs compared with BSPI by various spectral means characterization analysis. These favorable factors significantly enhanced the photocatalytic degradation of methyl orange (MO) by HSPI. This work provides a promising approach for the preparation of cheap, efficient, environmentally friendly, and sustainable photocatalysts.

## 1. Introduction

Recently, in the field of photocatalysis research, polymer semiconductor-based photocatalysts have always received attention because of their excellent physicochemical properties and stability [1,2,3]. Polyimide (PI), as a typical metal-free photocatalyst semiconductor with photocatalytic properties, has exhibited great promising potential for photocatalytic hydrogen production [4,5], carbon dioxide reduction [6], and organic pollutant degradation [7,8]. However, the inherent drawbacks of PI itself, such as low specific surface area, high charge carrier prohibition bandwidth, and low separation efficiency of photogenerated electron and hole pairs [9], have restricted the high-efficiency photocatalysis of PI for further applications. For this cause, Wang et al. modified polyimide with sulfur by element doping to improve its photocatalytic activity [10]. However, the bulk SPI (BSPI) with irregular block layer structure prepared by the conventional method still has some drawbacks, such as small specific surface area and low pore capacity, leading to finite reaction sites, and its photocatalytic activity needs to be further improved [11].

In order to increase the surface area, porosity, and photocatalytic activity of materials, great efforts have been made by researchers in designing and preparing composites with hollow shell structures [12,13] and heterojunctions [14,15,16]. In this regard, hollow structures provide favorable light absorption conditions due to their large specific surface area, porous spherical shells, and internal cavities. Therefore, the construction of the hollow shell structure provides another new approach to the modification of polymer structures. Methods such as the hard template methods [17,18] and soft template [19,20] are usually used to prepare the template material first, then the composite material is built on its outer surface, and finally, the template is removed. Many reports have been recently published on the use of hard or soft templates to modify the structure and porous morphology of graphitic phase carbon nitride (g-C_3_N_4_) [21,22,23,24,25,26]. In particular, the hard template approach using mesoporous SiO_2_ [27] has been widely used in g-C_3_N_4_ with similar structure and properties to BSPI. Hollow shell materials with porous structures exhibit a superior photocatalytic activity, which is attributed to their typical pore structure leading to a high surface area and excellent multiple light scattering properties [28,29,30], where rapid electron migration occurs through restricted pore channels under visible light. As far as we know, no relevant studies on the modification of BSPI with a hollow shell structure have been reported. Based on the above discussions, it is important to design and prepare SPI with a porous hollow shell structure for further applications in the field of photocatalysis.

In this study, we prepared highly crystalline sulfur-doped polyimide hollow nanoshell (HSPI) with controlled shell layer thickness using dense monodisperse nanostructured silica templates. Methyl orange (MO) is a common organic pollutant, non-volatile, with strong antioxidant and direct photodecomposition resistance [31], and is often used as a model pollutant for photocatalytic reactions. The photocatalytic performance of the prepared catalysts was evaluated by degradation experiments with methyl orange (MO) solution. Remarkably, HSPI with porous hollow spheres exhibited significantly enhanced photocatalytic activity for the degradation of MO solutions compared to BSPI. This may be attributed to its typical hole structure leading to high surface area and outstanding multiple light scattering properties, which greatly facilitate the effective separation of photogenerated electrons and holes and enhance the effective use of solar light. The possible mechanism of effective carrier separation and photocatalytic degradation of MO was also proposed.

## 2. Experimental Section

### 2.1. Synthesis of the Photocatalyst

Tetraethyl silicate (analytical purity), octadecyltrimethoxy silane (analytical purity), and ammonia (30–33%) were purchased from Shanghai Aladdin Biochemical Technology Co (China). Homothallic tetracarboxylic anhydride (99%) and melamine (99%) were purchased fromShanghai Maclean Biochemical Company (China), and sublimated sulfur (analytical purity) and ammonium hydrogen fluoride (analytical purity) were purchased from Tianjin Damao Chemical Reagent Factory (China).

The synthesis of sulfur-doped polyimide was based on that reported by Ma et al. [11]. Specifically, a certain amount of melamine, homothallic anhydride, and sublimated sulfur was weighed and dispersed in an aqueous ethanol solution after thorough grinding of the three, loaded into an inclined four-necked flask for vacuum stirring, and then centrifuged and dried to obtain a solid, which was heated from room temperature to 325 °C at a heating rate of 7 K/min, and the BSPI was obtained after maintaining this heating temperature for 4 h.

The preparation of the porous hollow shell SiO_2_ template, according to the Stöber method [32] using tetraethoxysilane, is shown below. Specifically, mix 3.10 mL ammonia water (31 wt%) with 58.5 mL absolute ethanol and 10 mL deionized water, add 5.6 mL TEOS in the stirring state, and let it stand for 1 h. Prepare the mixture of TEOS and C_18_TMOS in proportion, add the above solution, disperse uniformly, centrifuge, and dry (70 °C); then, slowly increase the temperature from 25 °C to 325 °C at a heating rate of 7 K/min and maintain this temperature for 4 h. After heating, take it out after cooling, fully grind it, add 20 mL of 1 mol/L hydrochloric acid solution to remove unreacted impurities, and finally dry it overnight at 80 °C to obtain the silicon ball template. As the shell thickness is related to the addition amount of TEOS and C_18_TMOS [12], a series of TEOS and C_18_TMOS exploration experiments with different addition amounts were designed, as shown in Table S1.

**Scheme 1:**
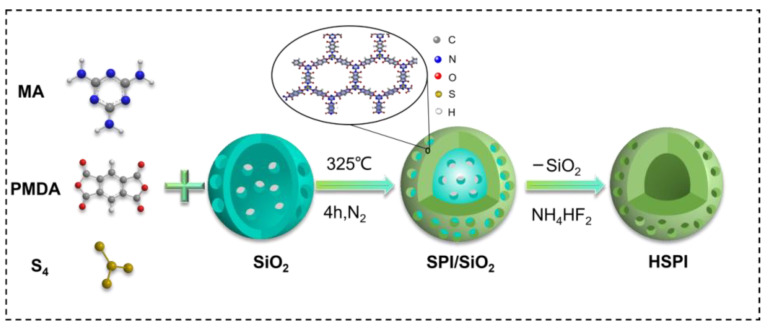
Schematic illustration of the fabrication process of HSPI.

HSPI was prepared using the following method. Typically, take 2 g of the prepared silicon ball template and grind it and mix it with 1.0 g of melamine, 1.7 g of pyromellitic anhydride, and 0.9 g of sublimed sulfur. After full grinding, dissolve it with anhydrous ethanol, disperse it with ultrasonic stirring, and centrifuge and dry it to obtain a solid powder. The hollow shell SPI was formed during the heat treatment at 325 °C. Subsequently, the silica template was removed by ammonium bifluoride solution to obtain the HSPI. The HSPI in the mesopore was synthesized using the simple heat treatment etching method shown in Scheme 1. In addition, monodisperse silica nanoparticles with different shell thicknesses of 130 nm, 120 nm, 100 nm, 90 nm, and 80 nm were used as templates to react with precursors of SPI, and finally, templates were etched to form sulfur-doped polyimide porous hollow shell materials of different thicknesses. The obtained samples are referred to as HSPI-x for short, where x stands for 1, 2, 3, 4, and 5, respectively.

### 2.2. Characterization

The X-ray diffraction patterns (XRD) of samples were obtained on an X-ray diffractometer with Cu Kα (λ = 1.540562 Ǻ) radiation in the 2θ range from 5° to 70°. Fourier-transformed infrared (FTIR) spectra were measured on a Nicolet 6700 spectrometer using the KBr pellet support. The scanning electron microscope (SEM) images were obtained by using a JSM-6610 system(HITACHI, Japan). The curves of the pore size distribution and surface area of the samples were inspected by Barrett−Joyner−Halina’s (BJH) method and Brunner–Emmet–Teller’s (BET) method, respectively. UV−vis spectra were tested on a Shimadzu UV−2600 spectrometer(Japan) and BaSO_4_ was used as a reference at room temperature. The photoluminescence (PL) spectrum was performed by an Agilent MY15170004 spectrometer (USA) with an excitation wavelength of 350 nm. X-ray photoelectron spectroscopy (XPS) and valence band X-ray photoelectron spectroscopy (VBXPS) were performed on a PHI 5000 Versa Probe X-ray photoelectron spectrometer (a monochromatized Al Ka X-ray radiation, ULVAC-PHl, Japan).

### 2.3. Photoelectric Chemical Measurements

The photoelectric chemical properties of the fabricated samples were measured in a conventional three-electrode system. It consists of an electrolyte solution (Na_2_SO_4_, 0.5 mol L^−1^, pH = 6.8), a counter electrode (a small piece of platinum), a working electrode, and a reference electrode (Ag/AgCl electrode). A transparent conductive film glass for the working electrode is usually fabricated on fluorine-doped tin oxide (FTO) using the electrophoretic deposition method. Typically, 10 mg I_2_ and 50 mg of powder sample were dispersed in 50 mL of acetone for 30 min under sonication to obtain a cloudy liquid. At the same time, two pieces of FTO glass were mounted in the electrophoretic deposition equipment submerged in a turbid liquid. The switch was turned on, and the voltage was kept at 20 V for 5 min. Thus, one piece of FTO is prepared on the conductive surface with a certain sample of glass and dried in air. In order to reduce the size of the light-irradiated area and prepare the sample layer, the light was irradiated on the exposed area of 0.28 cm^2^ and on the back side (FTO substrate/semiconductor interface). Electrochemistry was performed on an electrochemical analyzer (CHI-663C, Chen-Hua, Shanghai, China).

### 2.4. Photocatalytic Performance Tests

Photocatalytic degradation of methyl orange (MO) solution was used to determine the photocatalytic activity of the prepared samples. The light source is a 300 W xenon lamp (I = 20 a) with a cooling fan. The photocatalytic reactor used Pyrex (Perfectlight Technology Co., Ltd, Beijing, China) top irradiating vessel and a constant temperature water system, and the temperature was controlled at 25 °C. Typically, 0.2 g of catalyst was placed in a photocatalytic reactor containing 100 mL of methyl orange solution (400 mg/L). It was first allowed to adsorb statically under dark light conditions for 1 h. Then, the xenon lamp was turned on to give off conditions, and a certain amount of the reaction mixture (3–5 mL) was removed at regular intervals and centrifuged, and the absorbance of the resulting clarified solution was measured at 464 nm using a UV-Vis spectrometer (Mapada UV-1800, Shanghai, China).

## 3. Results and Discussions

### 3.1. Structure and Morphology Analysis

**Figure 1:**
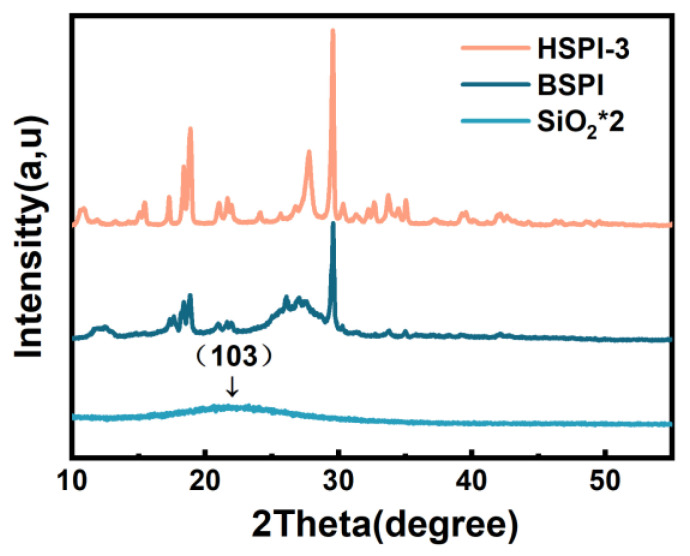
XRD patterns of the prepared SiO_2_, BSPI, and HSPI-3 samples.

X-ray diffraction (XRD) maps were used to characterize the composition and crystalline phase structure of the prepared samples. As shown in Figure 1, the X-ray diffractograms of the prepared SiO_2_, BSPI, and HSPI-3 samples display that the BSPI samples have obvious characteristic diffraction peaks at 18° and 29.5°, indicating that the S_4_, MA, and PMDA have been polymerized into the BSPI backbone [10]. The SiO_2_ prepared in this study belongs to amorphous crystals, and its crystal diffraction intensity is not obvious. After multiplying the XRD data of silica by 2, it can be seen that the XRD diffraction peak near the bun peak of 22° in SiO_2_ corresponds to (103) of SiO_2_. After the SiO_2_ template was etched, the peak corresponding to the SiO_2_(103) reflection disappeared in the HSPI-3 sample, signifying the absence of SiO_2_ in the final product [33]. Furthermore, the positions of the characteristic diffraction peaks of the HSPI-3 samples were not shifted compared to the BSPI, while the diffraction intensity was dramatically increased. This may be attributed to the fact that the precursor of SPI was more uniform as the SiO_2_ nanospheres and acted as a templating agent and dispersants, and the polymerization and crystallization of SPI were more sufficient during the thermal polymerization process. It is commonly believed that the highly crystallized photocatalyst can improve the migration of charge carriers due to the much denser packing of the layers and a smaller number of defects [34,35,36]. It indicates that the crystal structure of the HSPI-3 samples did not change, and the crystallinity is stronger [37].

**Figure 2:**
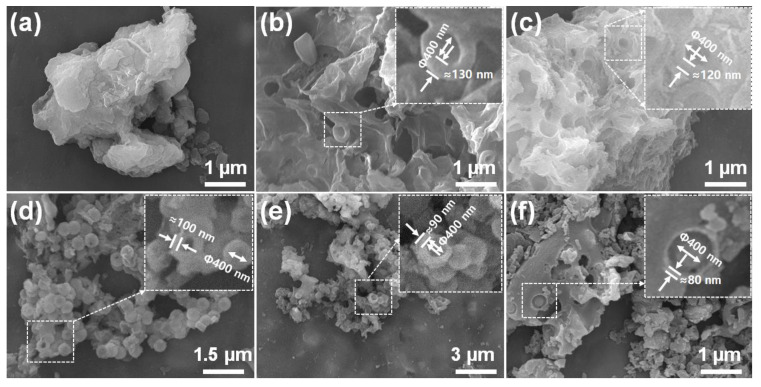
SEM of BSPI (**a**) and HSPI-1 to HSPI-5 (**b**–**f**) with different thicknesses.

**Figure 3:**
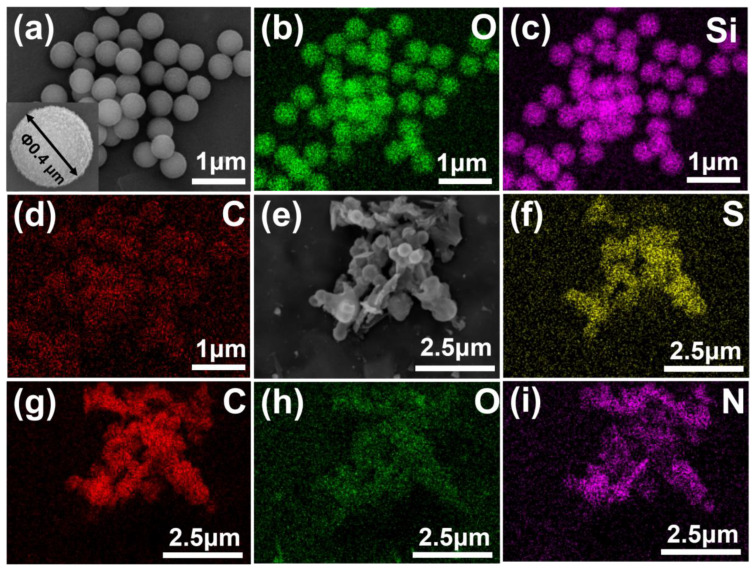
Elemental mapping images of SiO_2_ (**a**–**d**) and HSPI-3 (**e**–**i**) composite sample.

Figure 2 shows the scanning electron microscope (SEM) images of BSPI and different addition ratios resulting in different thicknesses of HSPI. As shown in Figure 2a, the BSPI is heavily agglomerated and shows a clear blocky accumulation form [11]. It is well known that the thickness of the shell layer of HSPI gradually becomes less as the proportion of silica template precursors increases (Figure 2b–f). In particular, the inset of Figure 2b–f shows that the HSPI-1 to HSPI-5 samples exhibit the structure of hollow shell spherical particles with thicknesses of 130 nm, 120 nm, 100 nm, 90 nm, and 80 nm, respectively. Additionally, their average internal cavity size of about 400 nm, which is consistent with the size of the prepared silica template, indicating that the template preparation was successful, which is consistent with the literature [12]. It is interesting to observe that the spherical structure of HSPI-4 and HSPI-5 in Figure 2e,f has collapsed and ruptured because of the strong shrinkage stress caused by the decomposition of the silica template by the etching of ammonium hydrogen fluoride, which caused some thin silica shells to collapse or break into pieces [38]. The morphology and microstructure of the as-prepared samples were characterized by EDX. As shown in Figure 3a–d, the EDX spectra of the template Si spheres samples show distinct signals of O, Si, and C elements, and the corresponding EDX mapping results confirm the synthesis of template SiO_2_ with a uniform distribution of elements.

**Figure 4:**
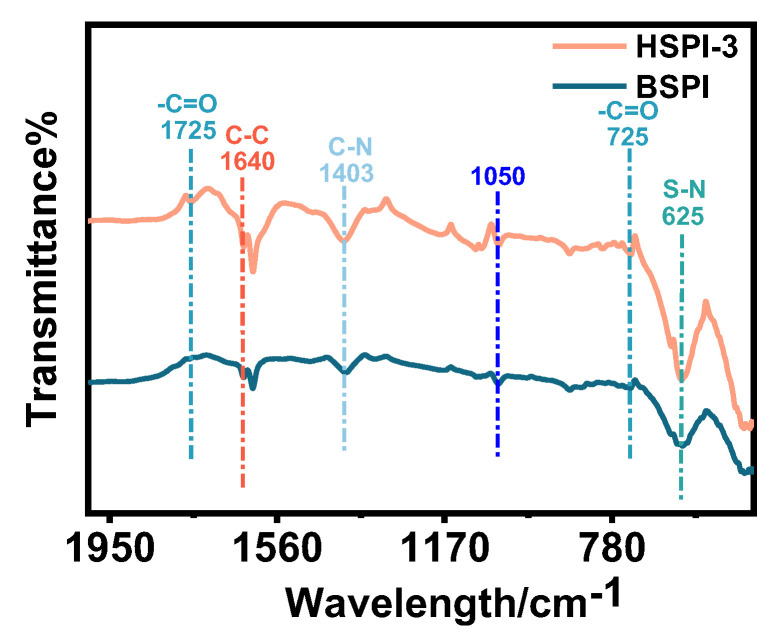
FT-IR spectra of HSPI-3 and BSPI sample.

In addition, the inset shown in Figure 3a is an SEM image of the template SiO_2_ spheres, which shows that the synthesized template SiO_2_ spheres possess a particle size of 400 nm and the surface of the spheres shows unevenness, which is attributed to the presence of mesopores or macropores on the surface of the prepared SiO_2_ spheres. The presence of these pores allows the transport of macromolecules in photocatalytic reactions [39]. Figure 3e–d shows the elemental distribution of the HSPI-3 sample, in which there are significant signals of N, O, S, and C elements, and no signals of Si elements are found. Moreover, the percentages of O and Si for SiO_2_ (in Figure S1a) and C, N, O, and S for HSPI-3 (in Figure S1b) in the elemental mapping are shown in Figure S1. Theoretically, the mass percentages of O and Si in SiO_2_ are 53 wt% and 47 wt%, respectively. However, the mass percentages of O and Si in the actually measured SiO_2_ are 71.58 wt% and 28.42 wt%, respectively, as shown in Figure S1a. This discrepancy may be due to the O_2_ in the air that inevitably remains on the surface of the SiO_2_ sample during EDS testing. Simultaneously, the mass percentages of C, N, O, and S in HSPI-3 are 43.76 wt%, 34.84 wt%, 18.32 wt%, and 3.08 wt%, respectively. These results also provide evidence of almost complete decomposition of the silica template. In addition, the FTIR spectra of BSPI and HSPI-3 samples are shown in Figure 4. 

**Figure 5:**
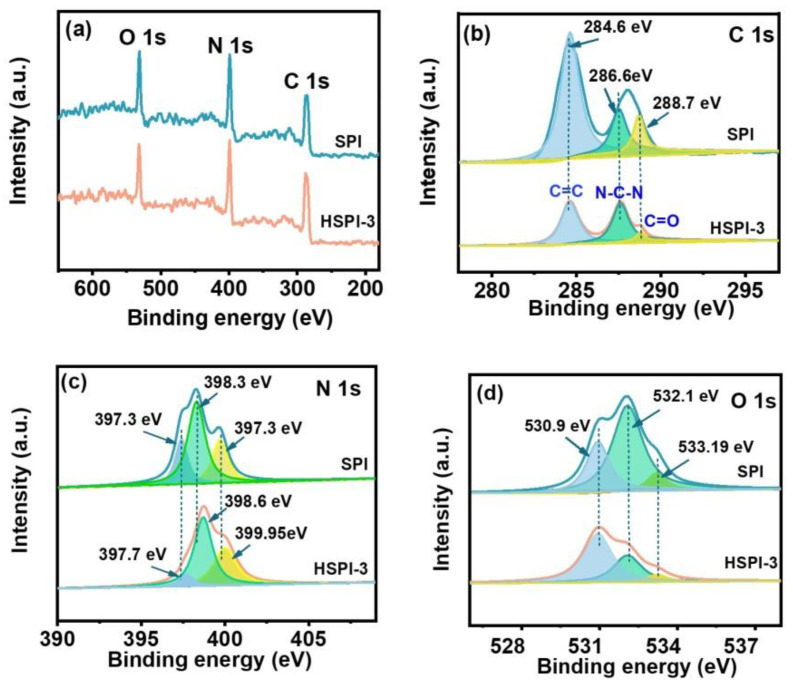
(**a**) XPS survey spectra for BSPI and HSPI samples; XPS spectra of (**b**) C 1s, (**c**) N 1s, and (**d**) O 1s of BSPI and HSPI-3 samples.

The peaks at 1725 and 725 cm^−1^ are attributed to the symmetric stretching and bending vibrational absorption peaks of –C=O on the five-membered acyl ring of BSPI, respectively. The absorption bands at 1640 cm^−1^ correspond to the aromatic C–C bonds of benzene rings in dianhydride blocks. Specifically, the aromatic C–C bonds of the benzene ring are some special bonds between single and double bonds, which are neither some simple double bonds nor some single bonds [10]. The peak located at 1403 cm^−1^ is attributed to the C–N stretching vibration absorption peak on the five-membered imine ring [40]. Furthermore, no anhydride absorption peak at about 1850 cm^−1^ was detected in the BSPI, indicating the completion of the amidation reaction. A wave number of 1050 cm^−1^ should correspond to the stretching vibration peak of the epoxy group [41]. Moreover, the characteristic vibrational peak of the S–N bond appears at about 625 cm^−1^. These results fully indicate that the BSPI structure was well synthesized and that the formation of the porous hollow shell structure of the HSPI samples did not cause any obvious changes in its chemical skeleton. In addition, the infrared characteristic absorption peaks of HSPI are significantly enhanced compared with that of BSPI, which corresponds to the enhanced crystallinity of XRD (in Figure 1). The surface chemical state and elemental species of the synthesized samples were also investigated using XPS, and the results are shown in Figure 5. 

The binding energies of all the elements are corrected by the C 1s peak at 284.6 eV, which is attributable to the sp^2^ C=C bond or the contaminated carbon [14]. During XPS sample preparation, some contaminated carbon components will inevitably be absorbed on the surface of the sample due to the sample’s contact with air. After careful analysis of the contaminated carbon adsorbed on the surface of the sample, the corresponding chemical states are usually C−C, C−O, and C=O. Among them, the chemical state of C−C is relatively stable, and the corresponding binding energy is 284.6 eV, which is equivalent to a stable internal standard in the sample. Figure 5a shows the four peaks attributed to C 1s, N 1s, and O 1s for BSPI and HSPI-3 samples. It shows that the surface of the BSPI and the HSPI-3 samples have similar chemical elements and states. As shown in Figure S2a, the C 1S curve shows three types of carbon species present in the HSPI-3 samples. The N 1S curve indicates that there are two nitrogen species in HSPI-3 samples, as displayed in Figure S2b. In addition, Figure S2c shows that there are three types of oxygen species in HSPI-3 samples. The corresponding high-resolution XPS spectra of C 1s, N 1s, and O 1s of the prepared samples are shown in Figure 5b–d. It is obvious that the C 1s XPS spectrum of BSPI shown in Figure 5b can be fitted to three peaks located at 284.6 eV, 286.6 eV, and 288.7 eV. The peaks centered at 286.6 eV and 288.7 eV can be attributed to the N–C–N bond and the C = O bond. The positions of these three peaks were almost unchanged in the sample XPS spectra of HSPI-3. From Figure 5c, three N 1s characteristic peaks of BSPI can also be observed at 397.3, 398.3, and 399.6 eV. The peak at 397.3 eV is from a pyridine-like nitrogen atom (N–C=N), and the two peaks at 398.3 and 399.6 eV can be attributed to the nitrogen atom attached to the two carbonyl groups (C = O) groups [11]. Additionally, the HSPI-3 samples all show a slight increase in the binding energy about N 1s with 397.7, 398.6, and 399.95 eV, respectively. The increase in N 1s binding energy for HSPI may be attributed to the formation of larger delocalized π-conjugated bonds by the lone pair electrons of N atoms in the hollow shell structure. The O 1s XPS spectra of BSPI shown in Figure 5d can be fitted to three peaks located at 530.9 eV, 532.1 eV, and 533.19 eV. The two fitted sub-peaks at 530.9 eV and 532.1 eV are attributed to the C=O bond and the C–O bond [42,43]. In contrast, none of the HSPI-3 samples showed any significant change in binding energy about O 1S.

**Figure 6:**
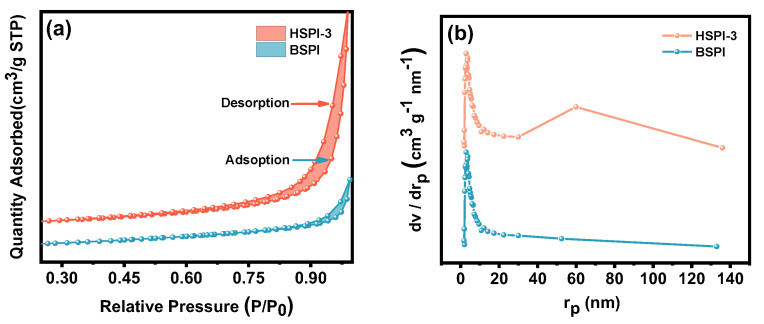
(**a**) Nitrogen adsorption–desorption analysis and (**b**) BJH plot for pore size distribution of BSPI and HSPI-3 composites.

The BET analysis was performed to verify the surface properties of the BSPI and HSPI-3 composite samples. As shown in Figure 6a, the specific surface area and porosity were determined using the N_2_ adsorption/desorption and Barrett–Joyner–Halenda (BJH) tests. According to the IUPAC classification, the BSPI and HSPI-3 composite adsorption–desorption curves could be classified as type IV [44]. A larger hysteresis loop can be found in the HSPI-3 sample, indicating a higher N_2_ adsorption capacity compared to BSPI. In addition, the BET surface areas of BSPI and HSPI-3 are 6.69 m^2^g^−1^ and 33.12 m^2^g^−1^, respectively. The specific surface area of the HSPI-3 sample is significantly higher than that of BSPI, which is almost five times larger than BSPI. In addition to the presence of mesopores in the range of 2–20 nm, such as BSPI, the HSPI-3 sample showed many large pores around 60 nm (Figure 6b). This would also provide abundant active sites for the photocatalytic degradation reaction, leading to high photocatalytic activity.

### 3.2. Optical Properties

**Figure 7:**
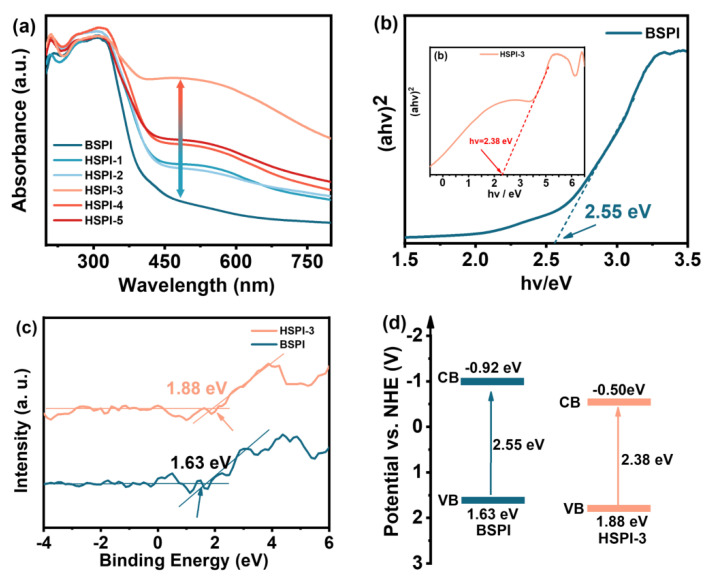
(**a**) UV–vis DRS spectra of BSPI and HSPI with different thicknesses. (**b**) The corresponding plots of (α·h*ν*)^2^ versus hν of BSPI and HSPI-3. (**c**) VBXPS plots of BSPI and HSPI-3. (**d**) Schematic illustration of band structures for BSPI and HSPI-3.

The optical absorption properties of the BSPI and HSPI-3 samples were characterized by UV-vis diffuse reflectance measurements. As shown in Figure 7a, it can be clearly observed that the optical absorption band edge of BSPI is about 450 nm, and the absorption in the visible range is relatively weak. Obviously, the HSPI samples show stronger spectral absorption relative to BSPI in the entire visible range. 

In particular, HSPI-3 exhibits the strongest visible light absorption intensity, which indicates that the HSPI-3 sample hollow shell nanospheres have the ability to reflect multiple times within the structure while increasing the effective path length of light absorption [45]. In addition, this enhanced light absorption in the visible range could also be due to an increase in the number of defects in the material when reducing the shell thickness to the nanometer range. In addition, the band gap of the photocatalyst can be evaluated by the Kubelka−Munk function equation, α·h*ν* = A (h*ν* − E_g_)^1/2^ [14]. As displayed in Figure 7b, the band gaps of BSPI and HSPI-3 samples are about 2.55 eV and 2.38 eV, respectively, by extrapolating the linear region of absorbance squared versus energy. Moreover, the VBXPS spectra of BSPI and HSPI-3 samples illustrated that the energy levels of the valence band (VB) in BSPI and HSPI-3 are approximately 1.63 and 1.88 eV, respectively, as displayed in Figure 7c. Thus, the conduction band (E_CB_) potentials are −0.92 eV and −0.5 eV for BSPI and HSPI-3 versus NHE estimated by the equation E_CB_ = E_VB_ − E_g_ [46]. Based on these results, the band structures of BSPI and HSPI-3 were determined, as shown in Figure 7d. It can be seen that the VB of HSPI-3 is lower than the VB of BSPI. It is further demonstrated that the HSPI-3 sample has a stronger oxidation capacity of the photogenerated hole and better stability compared to BSPI. This also indicates that catalysts with porous hollow shell structures have a narrowed band gap, thus enhancing light absorption and therefore generating more photogenerated electrons [47], which is beneficial for improving photocatalytic activity.

### 3.3. Photocatalytic Activity and Mechanism

In order to confirm the photocatalytic performance of the prepared samples, photocatalytic degradation experiments were conducted on the organic pollutant methyl orange (MO) solution (4 mg/L). As shown in Figure S1, because of the relatively stable structure of MO, the HSPI-3 sample could almost not degrade the MO solution even if the catalyst was placed under dark light conditions for degradation for 7 h. On the other hand, even under light conditions without placing photolysis of the MO solution was negligible in the case of photocatalyst placement.

**Figure 8:**
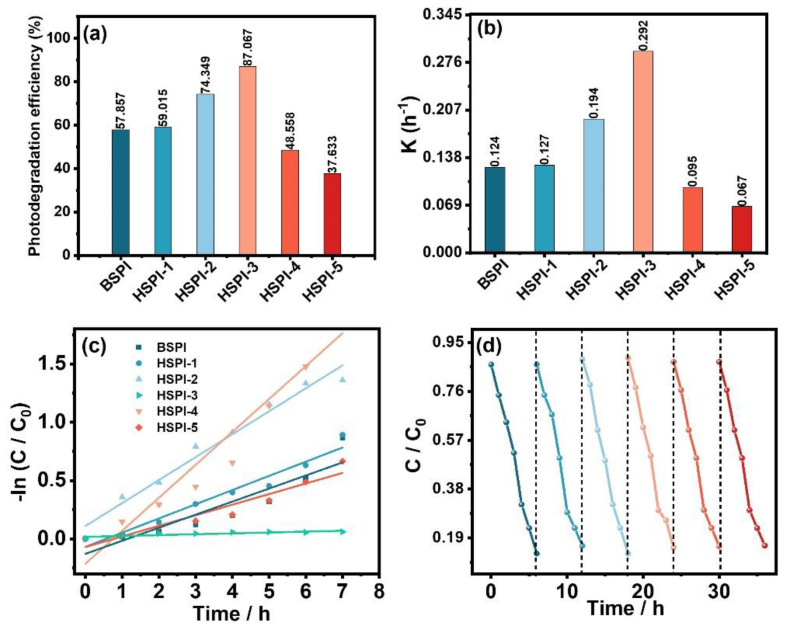
(**a**) Comparison of the photocatalytic degradation of efficiency MO over different catalysts under full arc light (λ > 300 nm). (**b**) The calculated correspondence of different systems. (**c**) Kinetic constants of MO degradation with different systems. (**d**) Cycling running for the photocatalytic degradation of MO over the HSPI-3 sample.

The photocatalytic activity of HSPI with different thicknesses for the degradation of MO solutions can only be achieved by adding a photocatalyst and under light irradiation.As shown in Figure 8, The activity obtained for these samples demonstrates that the degradation of MO solutions is driven by light energy. All HSPI samples exhibited different degrees of photocatalytic degradation of MO solution compared to BSPI. HSPI-3 showed the highest photocatalytic degradation activity, which can be attributed to the higher specific surface area and stronger light capture capacity of BSPI with a porous hollow shell compared to BSPI. In addition, the thickness of the hollow shell catalyst is another important factor affecting the photocatalytic performance of MO solution degradation. The HSPI-3 sample with 100 nm thickness not only shortens the light propagation path but also contributes to the mass transfer of reactants and products, thus increasing the carrier migration rate. Moreover, through UV-vis and fluorescence characterization, it was found that the HSPI-3 sample had the strongest visible light absorption and the weakest fluorescence intensity. These results indicate that HSPI-3 not only has the strongest light absorption capacity but also has a good separation efficiency of photogenerated electron–hole pairs and thus has the highest photocatalytic activity. The photocatalytic degradation efficiency of BSPI and HSPI samples for MO solution is shown in Figure 8a. It can be clearly seen that the HSPI-3 sample exhibits the highest MO photocatalytic degradation efficiency of 87.067%, which is about 150% higher than that of pure BSPI.

In addition, the reaction kinetics of the degradation of MO solution over BSPI and series HSPI catalysts were investigated, as shown in Figure 8b,c. It can be clearly seen that the linearly fitted data for the degradation of MO solution by a series of HSPI catalysts follow the first-order kinetics. Among them, the apparent rate constant K for porous hollow shell HSPI-3 is 0.292 min^−1^, which is about 2.36 times higher than that of bare BSPI (0.124 min^−1^). This result further confirmed the good photocatalytic performance of HSPI-3 for the degradation of methyl orange solution. This may be attributed to the improved charge carrier separation of HSPI-3 and the resulting higher photocatalytic efficiency. From Table S2, it can be seen that the R^2^ values for each sample are greater than 0.8, and the fit is good. However, the reaction has poor R^2^ values away from 1.0; thus, it may be a pseudo-first-order kinetic reaction. The stability test of the HSPI-3 sample for photocatalytic degradation of MO solution is shown in Figure 8d. The sample was tested after six cycles of 7 h each, which indicates the photocatalytic degradation performance of the HSPI-3 samples after light irradiation. The photocatalytic degradation performance of the HSPI-3 sample hardly diminished after 42 h and still had good photocatalytic activity, which indicates that HSPI has good stability.

**Figure 9:**
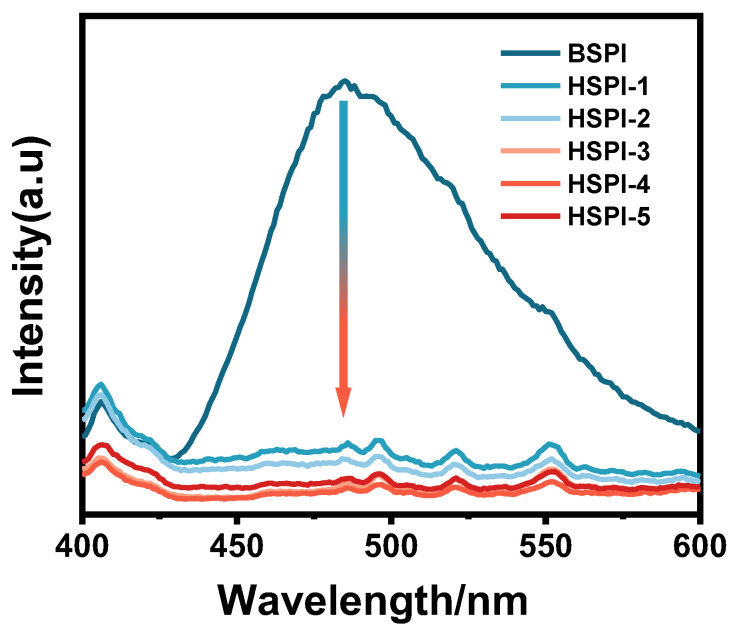
Comparison of photoluminescence spectra of the BSPI and HSPI samples.

Photoluminescence (PL) spectroscopy is conducted to evaluate the separation and complexation of photogenerated electron–hole pairs, which are critical factors affecting the efficiency of photocatalytic reactions. Figure 9 shows BSPI at an excitation wavelength of 350 nm and HSPI emission wavelengths with different thicknesses.

**Figure 10:**
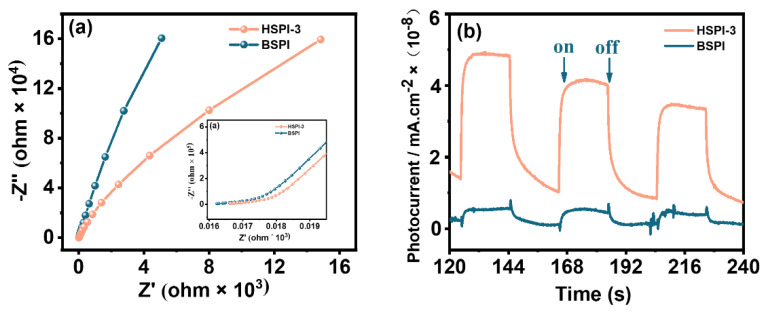
(**a**) EIS Nyquist plots of electrochemical impedance spectroscopy with BSPI and HSPI-3 composite in the dark. (**b**) Photocurrent–potential curves of BSPI and HSPI-3 composite electrode in the 0.5 mol L^−1^ Na_2_SO_4_ aqueous solution under full arc light irradiation.

Regarding BSPI, the strong emission peak at 485 nm was attributed to the band–band PL phenomenon with the energy of light approximately equal to the bandgap of BSPI (2.55 eV), which results from the n-π* transitions involving lone pairs of nitrogen atoms in the BSPI [48]. The PL curves of HSPI with different thicknesses have a shape very similar to that of BSPI. However, the intensity of the emission peak is dramatically reduced, indicating that the hollow shell structure of HSPI significantly suppresses the recombination of photogenerated electrons and holes. This is due to the fact that the outer space of the HSPI shell is separated from the inner space, making it possible to separate the charges in space. In addition, the thinner shell further shortens the diffusion path of electrons and holes and inhibits charge recombination [45], which facilitates the efficient separation of photogenic electron–hole pairs and enables further improvement of photocatalytic activity. Electrochemical impedance spectroscopy (EIS) measurements were performed to evaluate the interfacial charge transfer of the electrode materials. Figure 10a shows the EIS Nyquist plots of the BSPI and HSPI-3 samples in 0.5 M Na_2_SO_4_ electrolyte in dark light.

It can be observed that the two impedance spectra consist of semicircular arcs in the high-frequency range and straight lines in the low-frequency region. In particular, the inset in Figure 10a shows the Warburg behavior characteristic of the transmission line, which can be clearly observed in the high-frequency range of the EIS spectra of the BSPI and HSPI-3 samples [49]. As we know, the Warburg behavior is a diffusion resistance of electrolyte ions in the electrode material, which may be caused by the electrode surface roughness [50]. Apparently, the EIS Nyquist plot of the HSPI-3 sample has excellent conductivity compared to the BSPI in the non-photoexcited state. This may be attributed to the increased specific surface area due to the increased pore structure and surface roughness. Moreover, the equivalent circuit diagram is shown in Figure S4, where Rh is the impedance of the HSPI-3 material and the electrolyte solution, R1 represents the impedance of electron diffusion in the inner layer, R2 represents the impedance of electron diffusion in the outer layer, and CPE represents the double layer capacitance of the ohmic and Faraday processes [51]. In addition, as shown in Figure 10b, the photocurrents of the BSPI and HSPI-3 samples were stable and reproducible during several intermittent on–off visible light irradiation cycles. For the HSPI-3 sample, the photocurrent response is higher than that of BSPI (about five times higher than that of BSPI), thus indicating that the HSPI-3 sample has a higher electron–hole separation rate. These results suggest that HSPI catalysts with porous hollow shell structures facilitate the interfacial separation of electron–hole pairs, which allows for incident light reflection over longer distances. Thus, both the increased surface area and the porous structure favor multiple reflections of incident light [52]. 

**Figure 11:**
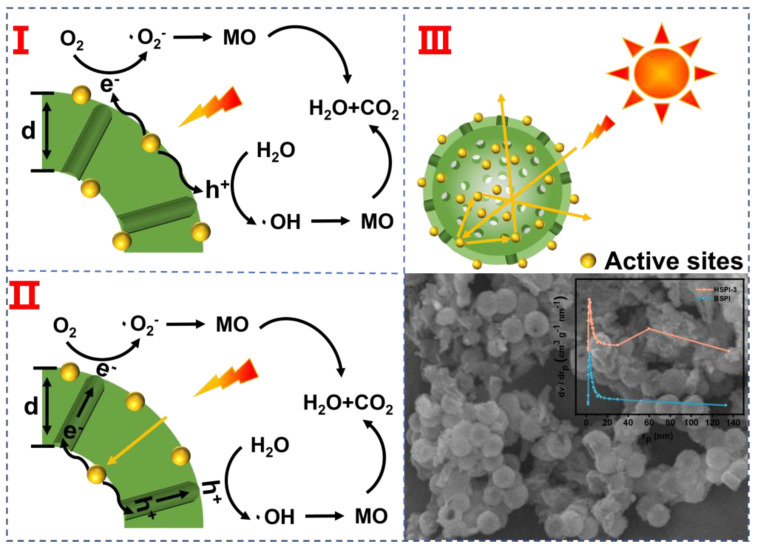
(**I**–**III**) Mechanism of photocatalytic degradation of MO by HSPI.

In this section, the possible separation process of electron–hole pairs in porous hollow shell catalysts under light conditions is proposed in-depth, taking HSPI-3 in this study as an example. As shown in Figure 11, HSPI-3 has a 100 nm thick shell space dividing it into two surfaces, the inner and outer, and thus the active site distribution range is significantly larger than that of the BSPI. 

Usually, the active sites are the surface of the photocatalyst, where superoxide radicals and hydroxyl radicals as active species oxidize MO to produce small inorganic molecules such as CO_2_ and H_2_O. In addition, the presence of surface mesopores makes it possible to connect two isolated spaces inside and outside. Therefore, the construction of this structure has a significant contribution to the improvement of photocatalytic activity. Specifically, as shown in Figure 11I, when the incident light irradiates the outer surface of the catalyst, the active sites on the outer surface were driven to generate photogenerated electron–hole pairs, thereby performing in situ degradation of the MO solution. On the other hand, it can be seen from Figure 11II that the incident solar light enters the inner surface of the catalyst through the shell layer thickness of 100 nm, driving the active sites on the inner surface to produce photogenerated electron–hole pairs, which are then transferred from the inner surface to the outer surface by the nearby mesopores to participate in the degradation of the MO solution. It makes the spatial separation of photogenerated electrons and holes possible and greatly reduces the complexation rate [45]. This can explain the sudden drop in fluorescence intensity in Figure 8. However, as can be seen in Figure 11III, the scattering of light and diffuse photon effect also occurs at the inner surface through the shell layer, which further improves the utilization of the solar light by the catalyst. Usually, the photogenerated electron (e^−^) capture oxygen molecules (O_2_) and form superoxide radicals (^•^O_2_^−^) [53]. Simultaneously, the hole (h^+^) reacts with H_2_O to produce hydroxyl radicals (^•^OH) [54]. As the photocatalytic active species, both ^•^O_2_^−^ and ^•^OH can easily oxidize MO to some small inorganic molecules such as CO_2_ and H_2_O [55,56]. The main reaction steps of the mechanism of photocatalytic degradation MO of prepared photocatalyst under light irradiation are summarized by the following Equations (1)–(5):

(1) $$\mathrm{HSPI}\overset{\;h\nu \;}{\to }\mathrm{HSPI}\left(e^{-}\right)/\mathrm{HSPI}\left(h^{+}\right)$$

(2) HSPI(e−)+O2→HSPI+O•2−

(3) HSPI(h+)+H2O→HSPI+O•H

(4) O•2−+MO→CO2+H2O

(5) O•H+MO→CO2+H2O

## 4. Conclusions

In summary, a highly crystalline HSPI photocatalyst with a porous hollow shell was prepared for the first time by the hard template method. Due to the unique structure of hollow shells in unique mesopores, the HSPI catalyst exhibits better catalytic capability than pure BSPI in simulating the degradation of organic pollutants under solar light. BET and UV-vis analysis confirmed that HSPI exhibits a larger specific surface area and abundant reactive sites with improved light utilization. In view of the scheme shown in Figure 11, the presence of mesopores provides a vehicle for the separation and transport of carriers from the inner surface to the outer surface of the catalyst, making it possible for the carriers to be transported over space. This study emphasizes the application of constructing materials with porous hollow shell structures in the field of photocatalysis.

## Data Availability

Data will be available on request.

## Supplementary Materials

The following supporting information can be downloaded at: https://www.mdpi.com/article/10.3390/polym15040903/s1, Table S1 Explore the experimental scheme. Table S2. The R2 values of the prepared samples. Figure S1. The percentage of O and Si for SiO_2_ (a) and C, N, O, and S for HSPI-3 (b) in the elemental mapping. Figure S2. (a) Fine-scanned XPS spectrum within C 1s region, (b) Fine-scanned XPS spectrum within N 1s region, and (c) Fine-scanned XPS spectrum within O 1s region of HSPI-3 sample. Figure S3. Photocatalytic activities of the MO degradation of BSPI and HSPI with different thicknesses under full-arc light irradiation. Figure S4. The equivalent circuit diagram of HSPI-3 sample
