# Supplementary material for: Controllable Preparation of Highly Crystalline Sulfur-Doped Π-Conjugated Polyimide Hollow Nanoshell for Enhanced Photocatalytic Performance
Source: Polymers (Basel). 2023 Feb 11;15(4):903. doi: 10.3390/polym15040903 (PMC9964284; doi:10.3390/polym15040903)
Supplement: Supplementary file 1 [file polymers-15-00903-s001.zip › polymers-2141688-supplementary.pdf]

Controllable preparation of highly crystalline sulfur-doped π-conjugated polyimide hollow nanoshell for enhanced photocatalytic performance

Duoping Zhang [a], Chenghai Ma [a,*], Pei-dong Shi, Zuan Yang, Tongwei Rong, Liurui Xiong, Wenhui Liao

School of Chemical Engineering, Qinghai University, Xining, 810016, China

* Corresponding author at: School of Chemical Engineering, Qinghai University, Xining, 810016, PR China.
E-mail address:chmaqhu@163.com (C. Ma).

Table S1 Explore the experimental scheme.

Sample	TEOS（ml）	C18TMOS（ml）	PMDA（g）	MA（g）	S4（g）
HSPIS-1	6.3	3.0	1.7	1	0.9
HSPIS-2	5.4	2.5	1.7	1	0.9
HSPIS-3	4.5	2.1	1.7	1	0.9
HSPIS-4	3.6	1.7	1.7	1	0.9
HSPIS-5	1.8	0.9	1.7	1	0.9

Table S2. The R^2 values of the prepared samples.

Samples*	R^2
BSPI	0.9238
HSPI-1	0.9312
HSPI-2	0.9590
HSPI-3	0.8449
HSPI-4	0.9668
HSPI-5	0.9298

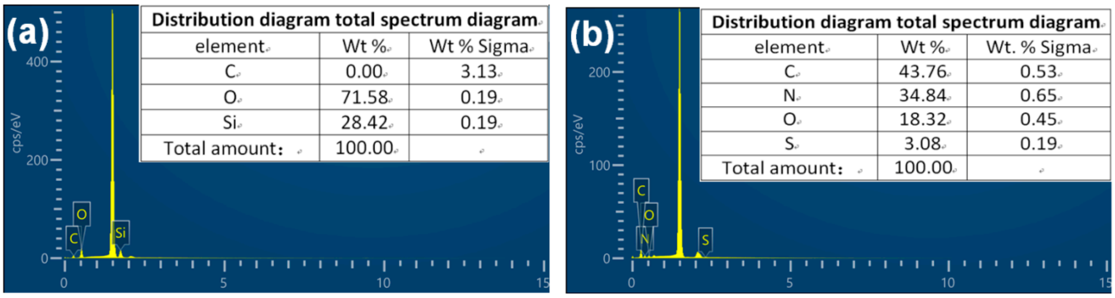

Figure S1. The percentage of O and Si for SiO_2 (a) and C, N, O, and S for HSPI-3 (b) in the elemental mapping.

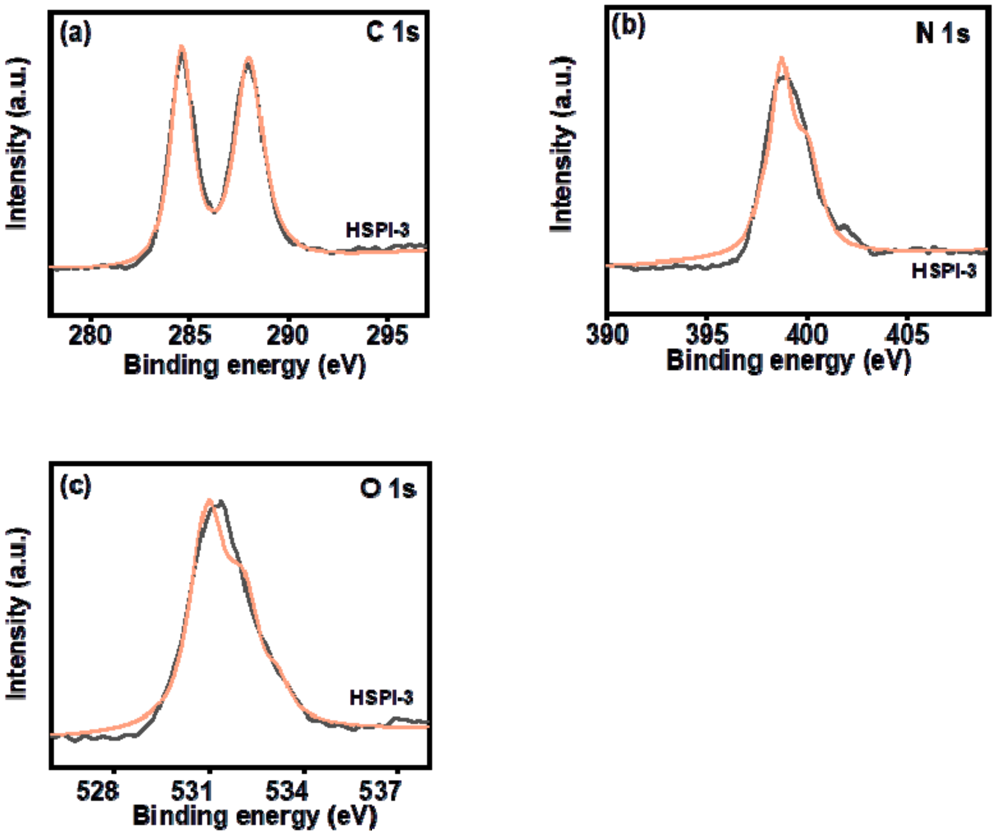

Figure S2. (a) Fine-scanned XPS spectrum within C 1s region, (b) Fine-scanned XPS spectrum within N 1s region, and (c) Fine-scanned XPS spectrum within O 1s region of HSPI-3 sample.

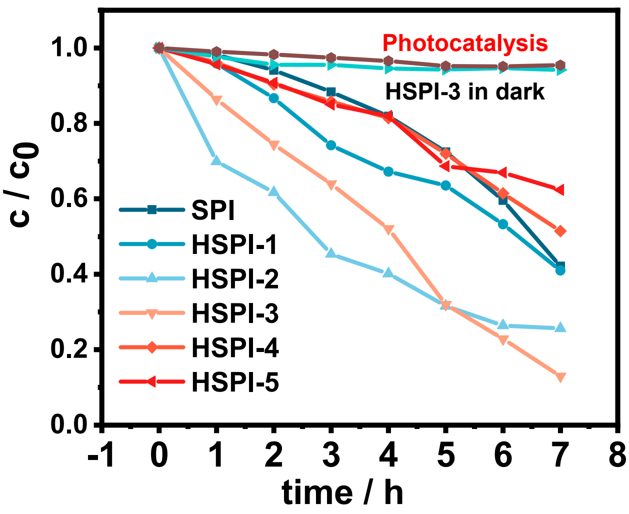

Figure S3. Photocatalytic activities of the MO degradation of BSPI and HSPI with different thicknesses under full-arc light irradiation.

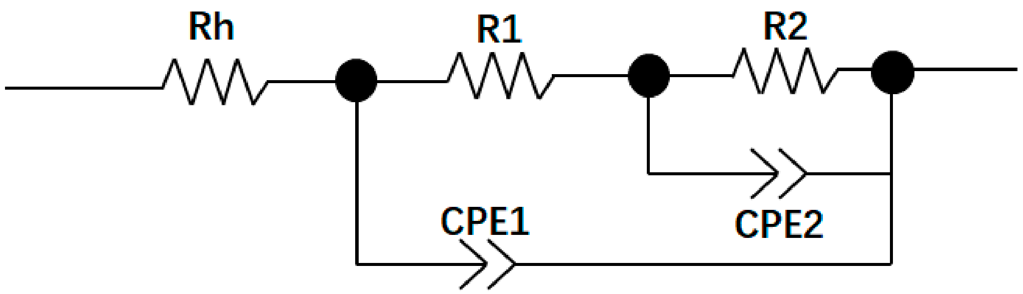

Figure S4. The equivalent circuit diagram of HSPI-3 sample.
